# Step by Step: Kinematics of the Reciprocal Trail Making Task Predict Slowness of Activities of Daily Living Performance in Alzheimer’s Disease

**DOI:** 10.3389/fneur.2018.00140

**Published:** 2018-03-14

**Authors:** Philipp Gulde, Katharina Leippold, Sarah Kohl, Timo Grimmer, Janine Diehl-Schmid, Alan Armstrong, Joachim Hermsdörfer

**Affiliations:** ^1^Department of Sport and Health Sciences, Technical University of Munich, Munich, Germany; ^2^Department of Psychiatry and Psychotherapy, Klinikum rechts der Isar, Technical University of Munich, Munich, Germany

**Keywords:** dementia, activity of daily living, assessment, kinematics, trail making task

## Abstract

Dementia impairs the ability to perform everyday activities. Reduced motor capacity and executive functions as well as loss of memory function and forms of apraxia and action disorganization syndrome can be reasons for such impairments. In this study, an analysis of the hand trajectories during the sequential movements in an adapted version of the trail making task, the reciprocal trail making task (RTMT), was used to predict performance in activities of daily living (ADL) of patients suffering from mild cognitive impairment and dementia. 1 patient with dementia of the Alzheimer’s type and 15 healthy, age-matched adults were tested in the standardized ADL of tea making and document filing. The characteristics of the kinematic performance in the RTMT were assessed, and models of multiple linear regression were computed to predict the durations of the ADL. Patients showed increased trial durations (TDs) in the ADL (Cohen’s *d*: tea making 1.64, document filing 1.25). Parameters and explained variability differed across patients and control as well as between different activities. The models for the patient sample were stronger and particularly high for the document filing task for which kinematics explained 71% of the variance ($R_{\text{adjusted}}^{\text{2}}$: tea making 0.62, document filing 0.71; both tasks combined patients 0.55, controls 0.25). The most relevant factors for the models were the TD and a parameter characterizing movement fluency and variability (“movement harmonicity”) in the RTMT. The models of multiple linear regression suggested that the patients’ activity of daily living performance was limited by cognitive demands, namely, identifying the varying targets during sequencing and the healthy controls’ performance by their motor capacity. Such models could be used to estimate the severity of ADL impairments in patients.

## Introduction

Dementia of the Alzheimer’s type [Alzheimer’s disease (AD)] and other types of dementia frequently impair independent living by decrements in the performance of activities of daily living (ADL) and even mild cognitive impairment (MCI) can lead to problems in complex ADL (1–3). Such activities can be basic hygiene, dressing, mobility/transfer, or food preparation (4, 5). Especially in AD, difficulties can arise from factors like an impaired motor capability (6–9), loss of focus (10, 11), and signs of apraxia and action disorganization syndrome (12, 13). The objective and quick assessment of ADL capability in a clinical setting is crucial for an effective treatment and support of patients (4). So far, clinicians score performance based on questionnaires and reports of patients and caregivers (1–5, 14, 15), or on observation and with timed tasks, e.g., trail making tasks (16–20). With motion tracking systems becoming affordable for clinical departments, the assessment can be enhanced to new technological standards, allowing more precise, objective, and less time consuming tests and patient classifications (21–23).

Trail making tasks are a common tool in clinical test batteries for the assessment of processing speed, mental flexibility, and executive functions (16, 24). Until now, the trail making tasks’ outcome parameters are the trial duration (TD) and the number of errors utilizing a stopwatch and a pen. Introducing motion tracking of the patients’ end effector would extend the possible quantification of performance by kinematic data, e.g., taking into account if a patient has severe motor impairments that are moderating the task performance. Further, it has been shown in stroke patients that the kinematic performance of complex tasks, in this case ADL, is influenced by the disease and can reveal impairments very specifically (25, 26). Since patients with MCI, dementia, and AD are also likely to reveal a decline in gross, fine, and complex motor function (7–9), the outcomes of trail making tasks might be influenced by this impaired motor function. So far, it has been shown that executive (dys)function and motor impairments are connected with ADL abilities in vascular dementia (27), and executive (dys)function and apathy are connected with ADL abilities in AD (28). In previous studies on aging (23) and stroke (26), we hypothesized that the sequencing of an ADL can be a demanding component that can impair performance. Therefore, abstract sequencing tasks could be strongly connected to ADL performance.

In this study, we introduced an adaptation of the trail making task A and examined the kinematics in the performance of 11 patients with AD and 15 age-matched control subjects. This reciprocal trail making task (RTMT) allowed a kinematic assessment of performance. In addition, to control for possible influences of impaired motor function we further applied a reciprocal aiming task (RAT), consisting of quick forth and back pointing movements between two marks, with emphasis on speed and accuracy. Patients and controls also performed two typical ADL that required the manipulation of everyday objects and which we have employed in previous studies in stroke patients: document filing (29) and making a cup of tea (26, 29). We hypothesized (1) that there are differences in the performance of the RTMT and the ADL between control subjects and patients, (2) that the performance in the RTMT could be a feasible tool for the assessment of the capability to perform ADL, and (3) that the RTMT is independent of the decline in pure motor capability associated with MCI, dementia, and AD.

## Materials and Methods

### Subjects

In this study, we tested a total of 11 patients with AD and 15 healthy, age-matched (*p* = 0.86) control subjects (Table 1), in 4 tests: the RTMT, document filing [activity of daily living and document filing (DF)] and tea making (activity of daily living and TM), and the RAT. The patient sample had an average age of 72.09a (controls 71.5a) and average Mini-Mental-State-Examination (MMSE) score of 23.6. Six of the patients showed symptoms of apraxia according to tests of apraxia [pathological scores in at least one of the tests: hand gesture imitation (score < 18/20), finger gesture imitation (score < 17/20), and pantomime of tool use (score < 45/55)] by Goldenberg (19). None of the controls showed signs of apraxia. Ethics approval was obtained by the ethics committee of the Faculty of Medicine of the Technical University of Munich. All participants gave written informed consent.

**Table 1:** Patients’ details including the patient # in the study, age, sex, diagnosis, count of pathologic scores in the three employed tests of apraxia, the score in the Mini-Mental-State-Examination (MMSE), and the performed tests.

Patient	Age (*p* = 0.63)	Sex (*p* = 0.36)	Diagnosis	Apraxia	MMSE	Performed tests
P1	77a	Male	AD (ICD-10: F00.2) Depression (ICD-10: F32.1)	1/3	23	RTMT, DF, and TM

P2	79a	Male	AD (ICD-10: F00.1)	0/3	19	RTMT, DF, and TM

P4	77a	Female	AD (ICD-10: F00.1) Depression (ICD-10: F32.1)	3/3	21	RTMT and TM

P5	81a	Male	AD (ICD-10: F00.1)	1/3	22	RTMT, DF, TM, and RAT

P7	73a	Male	AD (ICD-10: F00.1) Depression (ICD-10: F32.1)	2/3	22	RTMT, TM, and RAT

P9	76a	Female	AD (ICD-10: F00.2)	0/3	22	RTMT, DF, TM, and RAT

P10	85a	Male	AD (ICD-10: F00.1)	0/3	27	RTMT, DF, TM, and RAT

P11	68a	Female	AD (ICD-10: F00.1)	1/3	25	RTMT, DF, TM, and RAT

P12	57a	Female	AD (ICD-10: F00.0) Depression (ICD-10: F32.0)	0/3	28	RTMT, DF, TM, and RAT

P13	70a	Female	AD (ICD-10: F00.1) Depression (ICD-10: F33.1)	0/3	24	RTMT, DF, TM, RAT

P14	50a	Male	AD (ICD-10: F00.1) Depression (ICD-10: F32.1)	1/3	27	RTMT, DF, and TM

*n* = 11	72.09a ± 10.46a	6× Male 5× Female	11× AD 6× Depression	1× 3/3 1× 2/3 4× 1/3 5× 0/3	23.64 ± 2.84	11× RTMT 11× TM 9× DF 7× RAT

Control group (*n* = 15)	71.47a ± 6.23a	5× Male 10× Female	##	15× 0/3	##	15× RTMT 15× TM 15× DF 15× RAT

*The group did not differ in terms of age (*p* = 0.86) or sex (*p* = 0.279)*.

*AD, Alzheimer’s disease; MCI, mild cognitive impairment; FTD, frontotemporal dementia; RTMT, reciprocal trail making task; DF, document filing; TM, tea making; RAT, reciprocal aiming task; ICD; International Classification of Diseases*.

### Experimental Setup

**Figure 1:**
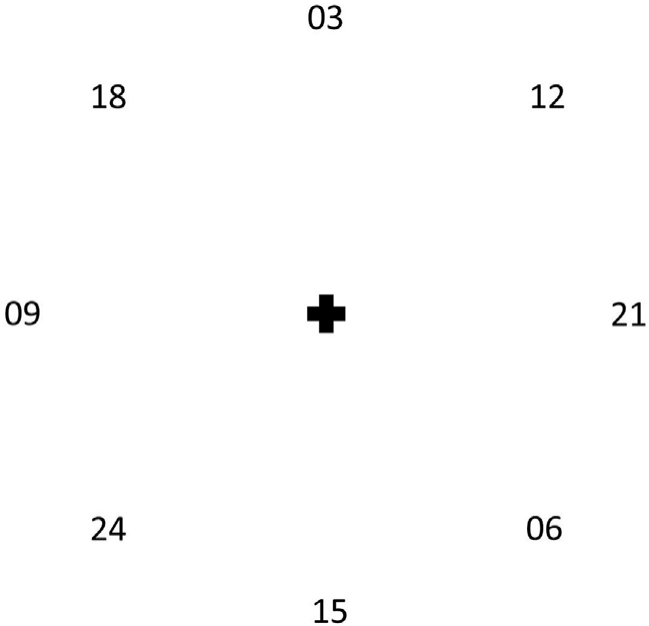
The working surface of the reciprocal trail making task. Participants were asked to start from the cross and move (index finger of the dominant hand) to the numbers in a rising order, returning to the cross after each movement, putting equal emphasis on speed and accuracy.

The RTMT (Figure 1) consisted of consecutive movements with the index finger of the dominant hand from a home position (cross) to eight numbers in rising order (steps of 3; 135° clockwise between following numbers). The participants were asked to place equal emphasis on speed and accuracy. The target numbers had a size of 1 cm × 1 cm and a distance of 8.5 cm to the home position. The RAT consisted of 30 repetitive movements of the index finger of the dominant hand between two marks sized 1 cm × 1 cm with a distance of 8.85 cm with equal emphasis on speed and accuracy. Due to errors in data management we only have data of 9× RAT.

The activity of daily living TM was to make a cup of tea with milk and sugar. The setup was the one described by Gulde and Hermsdörfer (23). The task was to prepare a cup of tea with milk and one sugar cube. Given objects were a mug and a tea spoon in front of the subjects. Further, in a semicircle from left to right: a water container, a milk carafe, a saucer for used tea bags, an open container with tea bags, an open container with sugar cubes, an open container with coffee powder as a distractor item, and an empty kettle. The activity of daily living DF was to file two sheets of paper in a cardboard file. The setup is the one described by Humphreys et al. (20), Bickerton et al. (18), and Bieńkiewicz et al. (29). Given objects were two sheets of DIN A4 paper (front, central), a cardboard file (back, central), a punch hole (left), and a stapler as a distractor item (right). A picture of the goal state of the task was shown to the subject prior to the start of the trial.

Participants first performed one trial of DF and then one trial of TM. They were asked to execute the tasks in a natural way, with no emphasis on speed. The outcome parameters of the ADL tasks were the TD (defined from video recordings) and the error frequency (see below).

To gather the kinematics of the index finger in the RTMT and the RAT, a passive marker of a Qualisys motion capturing system (Qualisys Inc., Gothenburg, Sweden), incorporating seven Miqus cameras at a frequency of 120 HZ, was attached to the dorsal part of the fingertip. The positional data were postprocessed using MatLab (MatLab R2015a, MathWorks, Natick, MA, USA). The raw data were filtered applying a 0.5 s local regression (“loess”) filter.

The following parameters were determined for each trial.

*TD*: time needed to execute the task in 1 s. In the case of tea making, the (passive) waiting time when boiling the water was excluded.

*Frequency*: movements per second in 1 Hz. A movement is defined by the transition from one mark to the other in the RAT.

*Relative activity (RA)*: relative amount of time with the velocity of at least one hand exceeding 0.05 m/s (23, 26).

*Path length*: length of the traveled trajectory in 1 m.

*Relative vertical path length*: path length in *z*-direction in relation to the total path length (23). This parameter describes one characteristic of the spatial composition of movements.

*Mean peak velocity*: mean of all velocity peaks with a prominence (intrinsic height) exceeding 0.05 m/s in 1 m/s. The mean peak velocity is used to display the general movement speed (23, 26).

*Relative number of velocity peaks*: the difference of the count of velocity peaks with a minimum magnitude of 0.05 m/s and the count of velocity peaks with a prominence exceeding 0.05 m/s in relation to the count of velocity peaks with a prominence exceeding 0.05 m/s as a measure of smoothness,

$$\text{Relative number of velocity peaks}=\frac{{\text{Peaks}}_{\text{Magnitude}}-{\text{Peaks}}_{\text{Prominence}}}{{\text{Peaks}}_{\text{Prominence}}}.$$

*Movement harmonicity (MH)*: each forth and back movement in the reciprocal tasks was extracted, and the velocity plotted against the position (phase plot). In a next step, the ratio of circumference and covered area of this ellipse-like plot (measured ratio) was set into relation to the ratio of circumference (30) and covered area of an actual ellipse with the maximum velocity and half of the movement amplitude as the radii (ideal ratio). The MH is the average of the differences of the ratios of these ellipse-like plots’ and the ellipses’ ratios divided by the ellipses ratio. The MH is employed as a measure of movement variability in movements. A related parameter called harmonicity has been published by Bieńkiewicz et al. (31). Values closer to 0 describe more harmonic movements,

$$\text{Movement Harmonicity}=\frac{\sum_{0}^{i}\left|\frac{{\text{IdealRatio}}_{i}-{\text{MeasuredRatio}}_{i}}{{\text{IdealRatio}}_{i}}\right|}{i},$$

$$\text{Ratio}=\frac{\text{Circumference}}{\text{Area}}.$$

*Errors*: number of performed errors. In TM and DF, the scoring was based on the errors classification by Hughes et al. (32). The scoring was done by a single, experienced rater. In RTMT, the classification is based on errors of omission (missing numbers), addition (additional numbers), and sequence (numbers in wrong order).

### Statistical Analysis

The statistical analysis consisted of the following: (1) *t*-tests between the groups and in case of significance the calculation of effect sizes [Cohen’s *d* (33)]; (2) a multiple linear regression to model the TD of the RTMT by the parameters of the RAT to check for an association between the motor capability and the performance in the RTMT for each group; (3) models of multiple linear regression, computed to model the mean *z*-scores (on the basis of the performance of the control group) of the TDs in the ADL (Combined = mean *z*-score of performance in the two ADL) for each group with the parameters of the RTMT, the MMSE score (in the patient group), the count of pathologic scores in the three tests of apraxia (in the patient group), and age; (4) models of multiple linear regression for the TDs of the two ADL (separately) and for the two groups to check for differences in the resulting equations. All multiple linear regression models were adapted stepwise by excluding non-significant factors and factors with a variance inflation factor (VIF) exceeding 5. α was set to 0.05. The threshold for collinearity was set to VIF > 5.

## Results

### Group Comparison

**Figure 2:**
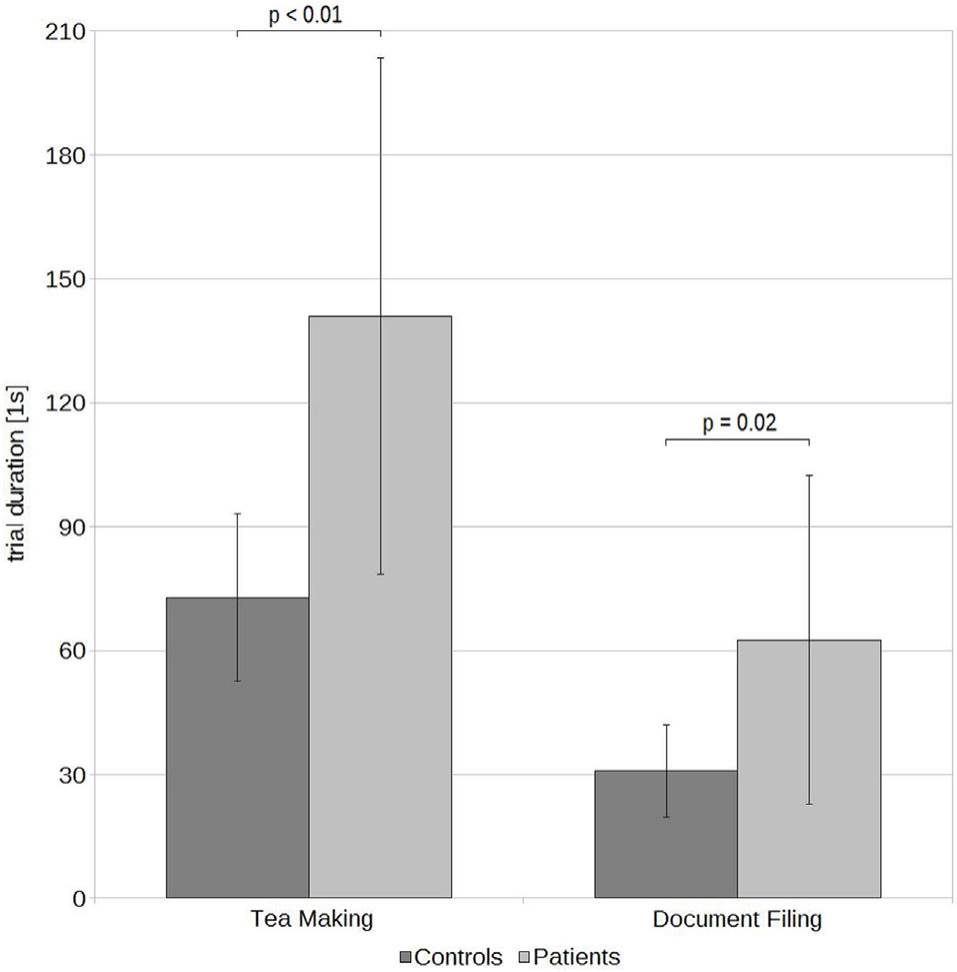
Mean trial durations and SDs of patients and controls in the two activities of daily living tea making and document filing.

**Figure 3:**
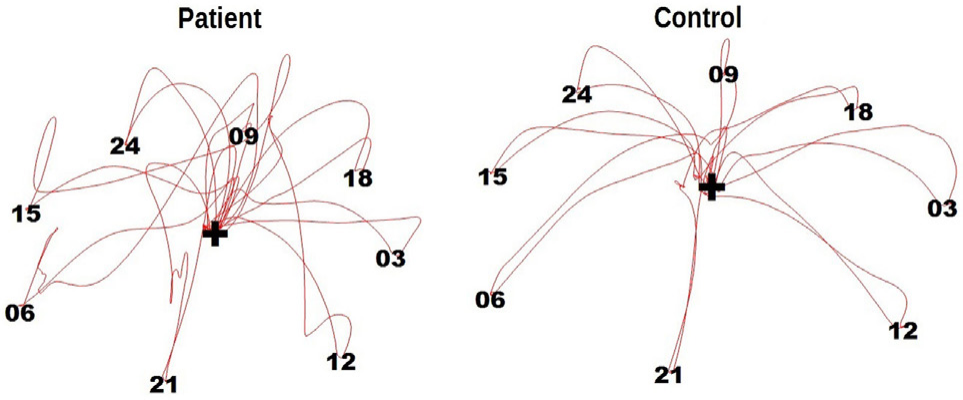
Exemplary trajectories of a patient (left) and a control subject (right) performing the reciprocal trail making task. The red lines indicate the movement of the fingertip in three-dimensional space.

**Figure 4:**
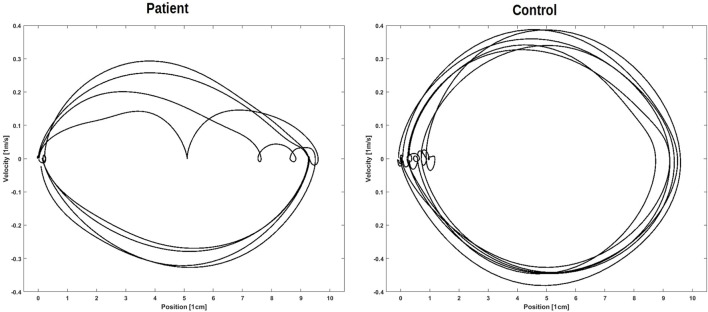
Corresponding phase plots. Such phase plots are the basis of the calculation of movement harmonicity. Left: display of four of one patient’s movements. Right: display of six of one control’s movements.

Two trials of DFin the patient sample were excluded due to the patient failing to execute the task (1× DF) or due to a TD being recognized as an outlier (1× DF,). The patients took an average of 62.6 ± 39.8 s (range: 20–132 s) for DF and an average of 141.0 ± 62.51 s (range: 69–260 s) for TM. The control had average TDs of 30.8 ± 11.2 s (range: 17–55 s) for DF, and 72.9 ± 20.3 s (range: 46–110 s) for TM (Figure 2). The means and SDs for the measures characterizing the performance of RTMT and RAT and the number of errors and TDs of DF and TM are displayed in Table 2. Note that only one of the patients was able to perform the RTMT without errors and only two of the controls committed an error in the RTMT. The most common error in the patient group was omission (0.73 ± 0.47), followed by addition (0.45 ± 0.69), and order (0.18 ± 0.40). The number of iterations (steps) in the RTMT did not differ between patients and controls (*p* = 0.64). All effect sizes in the kinematic parameters of the RTMT were in the range of 1.24–1.26. Exemplary trajectories in three-dimensional space of a patient (left) and a control subject (right) performing the RTMT are displayed in Figure 3. Figure 4 shows the corresponding phase plots, which are the basis of the calculation of MH.

**Table 2:** The means, SDs, *p*-values, and effect sizes (Cohen’s *d*) of the applied kinematic/non-kinematic parameters in the four tests.

	RTMT	RAT	TM	DF
*Trial duration (1 s)*	*p* = 0.03 Cohen’s *d* = 1.26	##	*p* < 0.01 Cohen’s *d* = 1.64	*p* = 0.04 Cohen’s *d* = 1.25
Patients	26.02 ± 15.64	##	141.00 ± 62.51	62.56 ± 39.78
Controls	13.48 ± 4.26	##	72.87 ± 20.33	30.80 ± 11.20

*Frequency (1 Hz)*	##	*p* = 0.59	##	##
Patients	##	3.42 ± 1.20	##	##
Controls	##	3.12 ± 1.14	##	##

*Relative activity*	*p* = 0.17	##	##	##
Patients	0.67 ± 0.24	##	##	##
Controls	0.78 ± 0.11	##	##	##

*Path length (1 m)*	*p* = 0.29	*p* = 0.63	##	##
Patients	3.46 ± 1.97	3.79 ± 0.29	##	##
Controls	2.76 ± 0.81	3.72 ± 0.41	##	##

*Relative vertical path length*	*p* = 0.26	##	##	##
Patients	0.46 ± 0.12	##	##	##
Controls	0.52 ± 0.13	##	##	##

*Mean peak velocity (1 m/s)*	*p* = 0.01 Cohen’s *d* = 1.25	##	##	##
Patients	0.36 ± 0.06	##	##	##
Controls	0.44 ± 0.06	##	##	##

*Relative number of velocity peaks*	*p* = 0.01 Cohen’s *d* = 1.24	*p* = 0.36	##	##
Patients	0.47 ± 0.24	0.11 ± 0.15	##	##
Controls	0.24 ± 0.14	0.21 ± 0.38	##	##

*Movement harmonicity*	*p* = 0.06	*p* = 0.35	##	##
Patients	0.32 ± 0.11	0.08 ± 0.03	##	##
Controls	0.24 ± 0.06	0.10 ± 0.08	##	##

*Errors*	*p* < 0.01 Cohen’s *d* = 2.12	##	*p* = 0.02 Cohen’s *d* = 1.35	*p* = 0.22
Patients	1.36 ± 0.81	##	2.09 ± 2.12	0.80 ± 1.03
Controls	0.13 ± 0.35	##	0.33 ± 0.49	0.33 ± 0.62

*RTMT, reciprocal trail making task; RAT, reciprocal aiming task; DF, document filing; TM, tea making*.

#### Modeling

#### RAT by RTMT

The model of multiple linear regression for the TD of RTMT by the parameters of RAT was not significant in the patient group but was significant in the control group with an $R_{\text{adjusted}}^{2}$ of 0.46 (*p* < 0.01). The frequency in the RAT was the only factor in the model with β = −0.793 (*p* < 0.01).

#### Combined by RTMT

The models of multiple linear regression for Combined (mean *z*-scores of both ADL) by the parameters of RMT were significant for both groups (patients: *p* = 0.01, controls: *p* = 0.03) with an $R_{\text{adjusted}}^{2}$ of 0.55 for the patients and a corrected $R_{\text{adjusted}}^{2}$ of 0.25 for the controls. Both models included MH and the patient model additionally included the TD as factors. The impact of the factors in the patient model were TD β = 0.899 (*p* = 0.01) and MH β = −1.063 (*p* < 0.01) and in the control model MH β = 0.552 (*p* = 0.03).

The corresponding equations are as follows:

$${\text{Combined}}_{\text{patinets}}=0.176*\text{TD}-28.827*\text{MH}+7.03,$$

$${\text{Combined}}_{\text{controls}}=7.877*\text{MH}-1.894,$$

where Combined = predicted mean *z*-score of the TDs in the ADL (TM and DF), TD = dimensionless TD of the RTMT, and MH = dimensionless MH in the RTMT.

**Figure 5:**
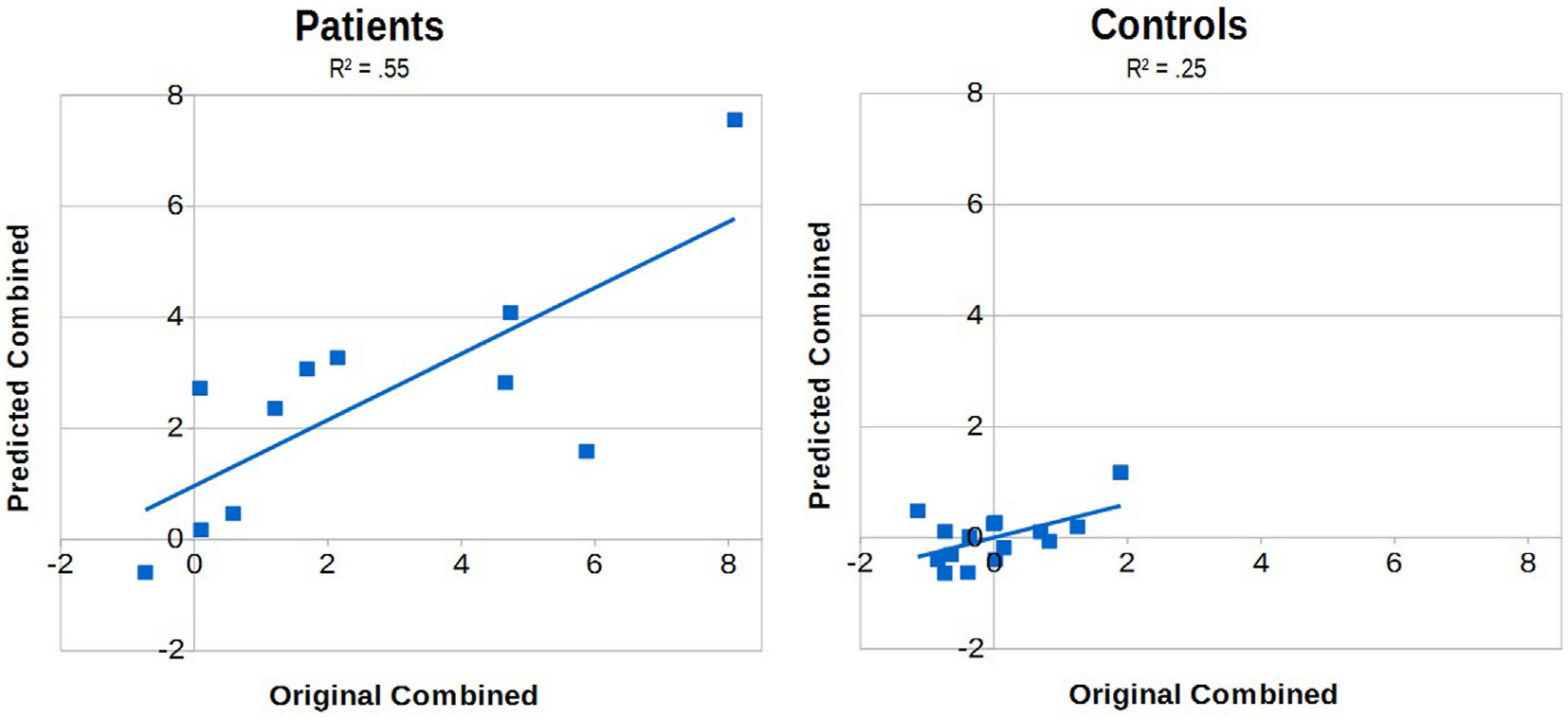
The predicted mean *z*-scores of the activities of daily living (predicted Combined) plotted against the mean *z*-scores of the original data (original Combined) for the patient sample (left) and the controls (right). The models of multiple linear regression were significant with *p* = 0.01 and an $R_{\text{adjusted}}^{2}$ of 0.55 for the patient sample and with *p* = 0.03 and an $R_{\text{adjusted}}^{2}$ of 0.25 for the controls. Note that none of the patients’ predicted scores was in the upper-left or lower-right quadrant.

The correlations of the predicted values against the original values are displayed in Figure 5 (left: patients; right: controls).

#### TM and DF by RTMT

The models of multiple linear regression for the two tasks in the two groups revealed differences concerning the significant factors (Table 3). In both patient models TD had a positive impact, being twice as strong in TM as in DF (TM β = 1.100, DF β = 0.603). In TM, the patient model additionally included MH (negative). There was no significant model in the control group for TM. The model for DF in controls had impacts of the relative number of peaks (smoothness) and RA (negative), while the patient model revealed a negative impact of the number of errors in the RTMT. Besides TD, there was no parameter overlap in both patient models.

**Table 3:** The different models of multiple linear regression for the two tasks in the two groups.

Group	Task	Model		TD		MH		Errors		rNP		RA	
$R_{\text{adjusted}}^{2}$	*p*	β	*p*	β	*p*	β	*p*	β	*p*	β	*p*
Patients	DF	0.71	<0.01	0.603	<0.01	##	##	−0.820	0.01	##	##	##	##
	TM	0.62	<0.01	1.100	<0.01	−0.965	<0.01	##	##	##	##	##	##
Controls	DF	0.43	0.01	##	##	##	##	##	##	0.725	<0.01	−0.703	<0.01
	TM	n.s.	##	##	##	##	##	##	##	##	##	##	##

*Note that there was no significant model for TM in the control group*.

*DF, document filing; TM, tea making; $R_{\text{adjusted}}^{2}$, the adjusted coefficient of determination; TD, trial duration; MH, movement harmonicity; Errors, number of errors in the reciprocal trail making task; rNP, relative number of peaks (smoothness); RA, relative activity*.

## Discussion

In this study, we examined the performance of 11 AD patients and 15 healthy, age-matched controls subjects in 2 ADL (TM and DF), the RAT (maximum motor capacity), and the RTMT (abstract sequencing task). Compared with healthy control subjects, patients revealed increased TDs in both tasks by approximately 100%. Increases of TD in the tea making task have already been observed in healthy elderly (+50% in comparison with young participants) (23) and in stroke patients (+50% in comparison with healthy, age-matched controls) (26). The kinematic performance in the RAT was comparable between the patient and the control group, while it strongly differed in the RTMT. Patients’ TD in the RTMT almost doubled, the mean peak velocity (general movement speed) was decreased, and the relative number of peaks (movement smoothness) was increased. Also, the error frequencies were increased in the RTMT as well as in TM, but not in DF. It appears that in this sample, the maximum motor capacity did not differ, in contrast to common findings (8, 9), but the performance in the ADL and RTMT did. Interestingly, the error frequency was only increased in TM and not in DF. In the stroke sample of Bieńkiewicz et al. (29), patients revealed comparable error frequencies in these two tasks, independent of the side of brain damage. But according to their findings, patients revealed different error types, with increases in conceptual errors and decreased spatiotemporal errors in TM in comparison with DF. Considering comparable maximum motor capacity, the low error frequencies in DF in our patient sample could be explained by this comparable motor capacity. Of cause these conclusions have to be taken with caution, since the error frequencies were assessed by a single rater. The analysis of error frequencies in the RTMT revealed that only one of the patients was able to perform the task without errors, while only two of the controls committed an error in the task. In addition, the most common error was the omission of steps, which would be typical for action disorganization syndrome (29, 34, 35). On the one hand, there was no association between the RAT and the RTMT in the patient group. Hence, kinematic changes in RTMT appear to be driven by cognitive demands, rather than maximum motor capacity. In the control group, on the other hand, there was a strong connection, with higher frequencies in the RAT leading to shorter TDs in the RTMT, indicating that the performance of controls in the RTMT is driven by maximum motor capacity.

The models for Combined by the kinematic parameters of the RTMT revealed strong models for each group. The model for the control group had an $R_{\text{adjusted}}^{2}$ of 0.25 and the model of the patient group an $R_{\text{adjusted}}^{2}$ of 0.55. The resulting prediction for the patient group notably revealed no false predictions in terms of the resulting quadrants (four quadrants: original +/− times predicted +/−, and false predictions would be combinations of + and −). The included factors in the two models were the MH (both models) and the TD (patient model). Interestingly, the impact of MH was positive in the control group’s model (less harmonic movements lead to higher TDs in the ADL), but negative in the patient group’s model (less harmonic movements lead to lower TDs in the ADL). Taking into account that the performance in the RTMT is influenced by the maximum motor capacity in controls, but not in patients, the underlying mechanisms may differ. In the control group, the harmonicity could be an indicator of certainty (e.g., less searching movements with the finger necessary in the RTMT), while in the patient group such movements could be able to support keeping the focus, which might have been a strategy that is also used in the ADL. The positive impact of TD in the patient model is indicating that prolonged TDs in the ADL are having a similar cause as in the RTMT.

The four models for the TDs of the two ADL in the groups differed substantially. On the one hand, the regression analysis for TM did not result in a significant model in the control group. This is interesting, since TM is the ADL with more degrees of freedom (more objects, more necessary action to achieve the task’s goal) and should therefore be more strongly associated with the cognitive demanding RTMT than DF. The model for DF in the control group was strong with an $R_{\text{adjusted}}^{2}$ of 0.43. Two factors were in the DF model: the relative number of peaks (smoothness) and the RA. Both parameters describe the capacity of motor planning [RA can be interpreted as accumulated reaction times (26)]. A reason could be the relatively small and complex objects (the lever arch system of the file and the punched holes in the sheets of paper) to handle in the DF. The variance in the TDs in DF in the control group seemed to be explained to approximately 43% by such capacities of motor planning.

The model for DF in the patient group was strong with an $R_{\text{adjusted}}^{2}$ of 0.71. The model had the factors TD and number of errors in the RTMT, indicating that the prolonged TDs in DF in the patient group had similar causes as in the RTMT. The missing impact of movement smoothness and RA in the patient model could be due to conceptual demands that limit performance (which would underline the impact of the error frequency in the RTMT). This has been shown in stroke patients (29). On the other hand, the model for TM in the patient group, being also strong with an $R_{\text{adjusted}}^{2}$ of 0.62, contained two factors: TD and MH. The impact of TD, again, indicates similar causes for the prolongation of the TD in TM. Interestingly, the impact of MH was, as in the combined model, negative, meaning that less harmonic movements lead to shorter TDs.

Interestingly, the sum of pathologic scores in the three apraxia tests was not included in the patient models. This could be due to apraxic behavior and resulting errors having a likelihood to be fatal. Future studies could employ logistic regressions to examine possible associations between task success and apraxia scores.

The present results indicate several meaningful points. AD had a strong impact on the performance of ADL. The performance in the RTMT differed between healthy controls and AD patients, and this difference appeared to be driven by the cognitive demands of the task for patients. The connection between ADL and RTMT performance is stronger in patients, which is underlined by the different impact of motor capacity on the performance in the RTMT. Analyses of the single ADL in both groups revealed that, if at all possible, predictions for controls are based on motor capacity. The patient models revealed some differences between the two ADL, showing that a generalization of ADL tasks should be done with caution. Nevertheless, TD was a common factor in both ADL models (and the model for Combined), leading to the assumption that the RTMT is covering similar cognitive demands as both tasks, which is very likely the sequencing. But one has to keep in mind that the sequencing in the RTMT is not driven by an ecological goal (like an end product in ADL) and the strong associations, especially in the patient group, could indicate different strategies of action planning in the two groups, meaning that patients appear to plan step by step, similar to a random walk. This could be tested by giving an ADL in which necessary objects are missing.

The neurological basis of the observed strong connection between executive functions (RTMT) and ADL could be of two kinds: impairments of synchronous processing or in visual scanning. Lafleche and Albert (36) suggested that a “concurrent manipulation of information” beyond a certain threshold of complexity, based on a degeneration of a “intracortical projection system,” could be an underlying neurological impairment, leading to decreased performance in tasks of executive functioning (e.g., trail making tasks) (36, 37). However, the impact rather lies in the trail making task B than A, due to the sequencing of numbers and letters (36). In the RTMT, this could be the sequencing of numbers and the return to the central mark that already exceeds such a complexity threshold. Looking at the tested ADL, it could be the mentioned (potential) step-by-step planning of the task that introduces a second information processing thread: the planning of the next step additional to the current object manipulation. Another potential explanation for the strong RTMT AD association could be the visual scanning capacity. It has been shown to impact trail making task performance (especially in type A) (38). In the tested ADL, patients were confronted with objects that can be slightly different from their domestic objects. A visual reassessment of their functioning and an adaptation to their handling could not only increase the visual demands but could also introduce a second processing thread to the patient.

The application of the RTMT as a clinical tool to assess ADL capability in patients seems to be promising. The model of Combined had no false positives or negatives in our sample. More interesting is the error frequency: only one of the patients was able to execute the task without errors and 13 of the 15 control subjects performed error-free. A combination of both approaches could include the following steps: (1) the error occurrence could indicate if the kinematic analysis is feasible. (2) The Combined model would then give an estimate of the severity of the possible ADL impairment (although we did not include fatal errors or failed trials in our analyses). These assumptions need further support by larger patient samples and a more comprehensive range of tested ADL.

## Ethics Statement

Ethics approval was obtained by the ethics committee of the Faculty of Medicine of the Technical University of Munich. All participants gave written informed consent.

## Author Contributions

PG and JH designed the study. SK, TG, and JD-S diagnosed and selected the patients. KL and SK organized the patients’ appointments and their transport. PG, KL, and AA performed the lab testing. PG performed the kinematic and statistical analyses. KL scored the ADL performance. All the authors contributed to the coordination of the study and the final draft of the manuscript.

## Conflict of Interest Statement

The authors declare that the research was conducted in the absence of any commercial or financial relationships that could be construed as a potential conflict of interest.

## Abbreviations

AD, Alzheimer’s disease; ADL, activities of daily living; DF, document filing; FTD, frontotemporal dementia; ICD, International Classification of Diseases; MCI, mild cognitive impairment; MH, movement harmonicity; MMSE, Mini-Mental-State-Examination; RA, relative activity; RAT, reciprocal aiming task; rNP, relative number of velocity peaks; RTMT, reciprocal trail making task; TD, trial duration; TM, tea making task.
